# Web-Based Motor Intervention to Increase Health-Related Physical Fitness in Children With Congenital Heart Disease: A Study Protocol

**DOI:** 10.3389/fped.2018.00224

**Published:** 2018-08-27

**Authors:** Michael Meyer, Adalheidur Hreinsdottir, Anna-Luisa Häcker, Leon Brudy, Renate Oberhoffer, Peter Ewert, Jan Müller

**Affiliations:** ^1^Department of Pediatric Cardiology and Congenital Heart Disease, Deutsches Herzzentrum München, Technische Universität München, Munich, Germany; ^2^Department of Sport and Health Sciences, Institute of Preventive Pediatrics, Technische Universität München, Munich, Germany; ^3^Costner, Reykjavik, Iceland

**Keywords:** web-based, intervention, eHealth, exercise, congenital heart disease, children

## Abstract

**Objective:** Exercise interventions are underutilized in children with congenital heart disease (CHD) especially when the primary outcome is not peak oxygen uptake. Most of the studies are restricted to a low sample size and proximity of the patients to the study centers. Now eHealth approaches bear a promising but also challenging opportunity to transmit such intervention programs to participants, and check progress and compliance from remote. This study will aim to improve health-related physical fitness (HRPF) with a 24 weeks web-based exercise intervention.

**Methods and Design:** The current study is planned as a randomized control trial (RCT) with a crossover design and the aim to improve functional outcome measures. It also estimates adherence and feasibility in patients with CHD in this web-based exercise/motor intervention over 24 weeks. Primary outcome will be the improvement of HRPF. Secondary outcomes are, functional and structural arterial stiffness measures and health-related quality of life. Thus, 70 children from 10 to 18 years with CHD of moderate and complex severity will be recruited and allocated randomly 1:1 in two study arms after baseline testing for their HRPF, arterial stiffness measures and health-related quality of life. For 24 weeks, participants in the intervention arm will receive three weekly exercise video clips of 20 min each. Every video clip comprises 20 child-oriented exercises which have to be executed for 30 s followed by a recovery period of 30 s. Each session will start with 3–4 warming-up exercises, followed by 10–12 strength and flexibility exercises, and ending with 3–4 min of cool down or stretching tasks. Continuous video clips will be streamed from a web-based e-Learning platform. The participant simply has to imitate the execution and follow some short advices. After each session, a brief online survey will be conducted to assess perceived exertion and feasibility.

**Discussion:** The study will help to determine the efficacy and applicability of a web-based exercise intervention in children with CHD in regard to functional outcome measures. In addition, it will outline the effectiveness of remote monitoring, which provides a cost effective approach to reach patients with CHD that are low in prevalence and often do not live in close proximity to their tertiary center.

**Trial Registration:** https://ClinicalTrials.gov Identifier: NCT03488797.

## Background

Children with congenital heart disease (CHD) show reduced motor competence or motor ability (1–4) as well as limitations in fine and gross motor skills (5–7). These skill-based limitations from early infancy are tracked into adulthood with further negative effect on health-related physical fitness (HRPF) and muscle strength (8–11). That those limitations still exist in the vast majority of children with CHD outline recent projects on health-related physical fitness (HRPF) and cardiovascular health (12–17).

These circumstances should in general be a reason to worry and a starting signal for exercise promotions and interventions to overcome such deficits to facilitate normal social integration and school sport participation as early as possible. Unfortunately, a review from 2013 outlines that exercise interventions are still underutilized, because of the relatively low prevalence of single types of CHD and the proximity to the study centers (18). One drawback is that the primary outcome of those studies always refers to improvements in peak oxygen uptake, while any forms of motor-related competences are of minor interest. This is probably the case, because motor-related competences do not represent a hard clinical endpoint like parameters derived from cardiopulmonary exercise testing (19–21). Another drawback refers to the willingness and availability of the parents to escort their children to the study centers.

Within the last years, digitalization has also shaped the field of medicine and many studies on eHealth and communication technology–based interventions for promoting physical activity (PA) show remarkable results (22). The new appealing possibility to transmit, conduct and control interventions from remote is also promising for studies in the field of patients with CHD. It is now possible to involve children with long distance to the study center to realize studies that otherwise would fail due to sample size. Currently children and adolescents grow up to be “digital natives” which are even more familiar in handling and executing app-based tasks, than dealing with paperwork. In addition, communication via social media platforms becomes more and more appropriate to monitor the study progress and maintain study compliance, instead of classic phone calls.

In patients with CHD, eHealth is largely unexplored with only one published study on physical exercise on aerobic fitness so far (23, 24). Therefore, we have launched a randomized controlled trial (RCT) that aims to improve functional outcome measures, primarily HRPF, in children and adolescents with CHD via a tailored web-based exercise/motor intervention over 24 weeks. That article describes the study protocol in detail.

## Methods

### Design and participants

**Figure 1:**
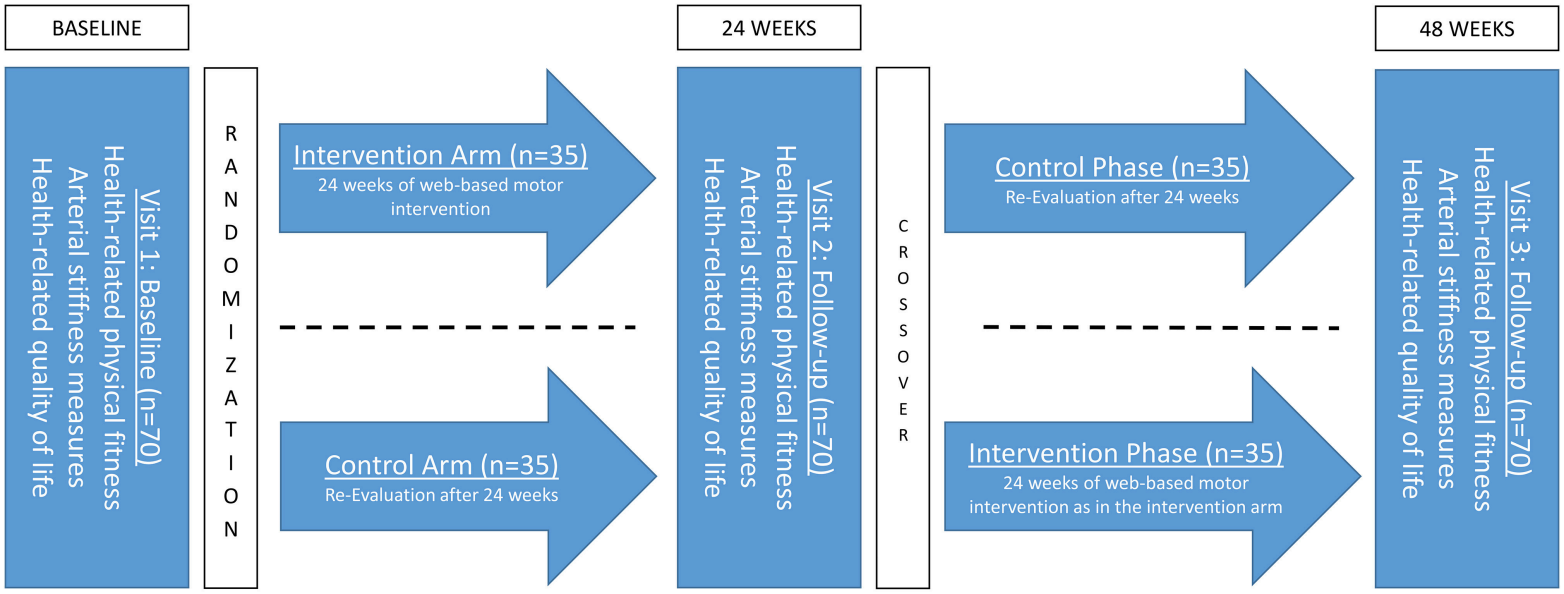
Study design and intervention scheme.

The current study is planned as a randomized control trial (RCT) with a crossover design and the purpose to improve functional outcome measures in patients with CHD via a web-based exercise/motor intervention over 24 weeks. The treatment scheme and data collection is presented in Figure 1. According to the inclusion and exclusion criteria outlined in Table 1, 70 children will be recruited and allocated randomly 1:1 in the two study arms after baseline testing. After 24 weeks, there will be a re-assessment of the clinical measurements.

**Table 1:** Inclusion and exclusion criteria.

**Inclusion criteria**	**Exclusion criteria**
Age 10–18 years old	Severe arrhythmias
CHD with moderate to complex severity according to the ACC criteria^*^	Severe left heart failure
Health-related physical fitness < 50th percentile (healthy reference)	Chromosomal anomalies and/or genetic syndromes
German speaking	Severe physical and/or sensory impairments (hearing, visual, or psychomotor)
Internet availability and an internet-capable device to use the intervention app	Elective cardiac intervention within the next 6 months following enrollment
Informed consent of parent/guardian as well as of the child	

* *According to Warnes et al. (25)*.

The accordance of the Declaration of Helsinki (revision 2008) and the Good Clinical Practice Guidelines is the basis of this upcoming study. The study protocol is already approved by the ethical board of the Technical University of Munich (project number: 130/17S). All children and their guardians will provide written informed consent. The trial is already registered at https://ClinicalTrials.gov (NCT03488797).

### Intervention

The motto of the exercise/motor intervention is “One Hour a Week, Brings Mobility, Power and Speed” which refers to the weekly training volume of 60 min. These 60 min are separated into 3 weekly sessions of 20 min each. The intervention is set up for 24 weeks (~6 month), resulting in a total of 72 exercise sessions (3 sessions per week over 24 weeks) for each participant.

Each session comprises 20 child-oriented exercises with the focus to increase strength and flexibility. Exercises are executed for 30 s followed by a recovery period of 30 s. Each session starts with 3–4 warming-up exercises, followed by 10–12 strength and flexibility exercises, and ending with 3–4 cool down or stretching tasks.

All exercise sessions are continuous video clips transmitted to the participants via the web-based e-Learning platform Einstein (https://einstein.costner.is). The participant simply has to imitate the execution and follow some short advices. For a better orientation, a timer in the upper right corner displays the duration of exercise and the time of recovery. Beep signals indicate the last 3 s of the exercise and recovery period.

After each session, the screen will jump to a short online survey. Participant will give a quick report on perceived exertion (Borg scale) and feasibility of the actual session. The investigator can thereby monitor adherence rate and, in case of lacking compliance, intervene via email or telephone calls. The three video clips will be available for 1 week (Monday to Sunday) and the participant is free to choose when to train. At the end of a week all sessions will be erased from the account and replaced by new 3 sessions. That procedure will be repeated over the target 24 weeks.

### Clinical measurements

All of the clinical measurements at baseline, short term (after 24 weeks of intervention and control group) and long-term (48 weeks after baseline) follow-up (Figure 1) will be conducted in the outpatient clinic of the German heart center in Munich.

#### Health related physical fitness (HRPF)

HRPF will be tested by five different tests of the FITNESSGRAM®(26) test battery in standardized order. The tests will be supervised by an experienced sports exercise physiologist.

- **Curl-Up**: Abdominal strength and muscular endurance
  Execution: Supine position, flexed knees with an angle of around 140° and feet placed on the ground. Arms stretched out beside the body. The upper body moves slowly toward the knees and afterwards back to the ground.
  Assessment: Score of valid curls-ups.
- **Push-Ups**: Upper body strength and muscular endurance
  Execution: Prone position, with stretched back and legs. Arms straight under the shoulders pushing the body up and down.
  Assessment: Score of valid push-ups.
- **Shoulder Stretch**: Upper arm and shoulder girdle flexibility
  Execution: Hands clenched to fists. Bring fists together as close as possible behind the back. Avoid hollow back.
  Assessment: Distance of the knuckles of the forefingers. Both sides.
- ∙ **Sit and Reach**: Hamstring flexibility
  Execution: Siting position, one leg stretched against a box, toes stretched upright. Other leg flexed. Reaching to the toes or further with straight arms.
  Assessment: Negative or positive values in cm depending on the zero line of the box/toes. Both sides.
- **Trunk Lift**: Trunk extensor strength and flexibility
  Execution: Lying outstretched in prone position. Arms tight to the body and hands under the thighs. Lifting upper body without bouncing while looking toward the ground.
  Assessment: Distance chin to ground in cm. Best of two trials.

Detailed information of the exercises and the test execution can be accessed from the online supplement of our recently published study in children with total cavopulmonary connection (13, 17).

#### Measures of arterial stiffness

##### Functional: pulse wave velocity (pwv) and central systolic blood pressure (CSBP)

Measures of arterial stiffness involve pulse wave velocity (PWV) and central systolic blood pressure. Functional measures of arterial stiffness play a significant role in the development of cardiovascular disease and associations to physical activity have been outlined in adult and pediatric cohorts as well (27, 28).

Therefore, the automated, oscillometric Mobil-o-Graph (I.E.M GmbH, Stolberg, Germany) is used. The measurement will be performed at the left upper arm after resting in supine position for 5 min. Cuff size is adjusted for individual arm circumference. The inbuilt ARCSolver algorithm of the device uses a transfer function to calculate PWV and central systolic blood pressure based on the peripheral wave form (29). The device has shown good validity and applicability in several studies, even in patients with CHD (12, 30, 31).

##### Structural: carotid intima-media thickness (cIMT)

Carotid intima-media thickness (cIMT) is a marker for structural changes of the vessels and for early atherosclerosis. It is shown that physical activity in general is associated with favorable IMT outcomes in children and adolescents (32, 33).

cIMT will be measured using B-Mode ultrasound. To minimize inter- and intra-observer variability the guidelines of the Cardiovascular Prevention Working Group of the Association for European Paediatric Cardiology are followed (34).

The semi-automated GM-72P00A Cardiohealth Station from Panasonic (Yokohama, Japan) is used together with a linear probe of 9 MHz to assess cIMT at the arteria carotis communis. The measurements are conducted in supine position with patients head turned 45 degrees to the opposite of the examined side and the neck slightly tilted backwards. In the first step, the neck vessels were scanned for plaques in a cross-section, afterwards the common carotid artery was displayed in the longitudinal view.

Pictures are then taken of the cIMT on the far-wall, in the end-diastolic phase, ~1 cm proximal to the bifurcation in two angles on the left (210° and 240° degrees) and two angles on the right side (120° and 150° degrees).

#### Health related quality of life (HRQoL)

The KINDL-R questionnaire is handed out to assess Health related Quality of Life (HRQoL). It is a common, international and well standardized questionnaire for evaluating children's HRQoL from a subjective perspective (35–38). It exists in three versions according to the different age groups. In this study, the KidKINDL for children aged 7–13 years and the KiddoKINDL for chidlren aged 14–17 years is used.

The questionnaire consists of 24 items that refer to the past week and is answered on a 5-point Likert scale (never, seldom, sometimes, often, and always). The scored items are then transferred to a total HRQoL score and to six subscales (physical, emotional, self-esteem, family, friends, everyday functioning). All subscales are graded on a scale from 0 to 100, whereby higher values reflect better HRQoL.

### Enrollment

The participants will perform the five motor tasks initially. Based on the test results of a reference cohort, LMS values were calculated according to Cole (39) using R-Studio (version 0.99.879, R-Studio Inc.) with the module extensions *gamlss* (version 3.4-8) and AGD (version 0.34). Children with CHD will be classified according to those established LMS values and z-scores displayed for every of the five tasks and HRPF z-score as the mean of the five tasks.

Participants are consecutive randomized and, when admitted to the intervention arm, start directly the week after screening for baseline characteristics with the exercise program. Every participant receives an anonymous account where 3 sessions will appear every week.

### Endpoints

#### Primary endpoint

The primary endpoint criterion refers to an improvement of health-related physical fitness, assessed as the mean z-score of the five tests, 24 weeks (~6 month) after intervention.

#### Secondary endpoints

Secondary endpoints are as followed:

- Compliance (adherence) with the supervised web- and home-based intervention measured as participation rate in the training sessions (%).
- Improvement of Central/peripheral blood pressure after 24 weeks (~6 month) after intervention.
- Improvement of Intima-media thickness after 24 weeks (~6 month) after intervention.
- Improvement of health-related quality of life 24 weeks (~6 month) after intervention.
- Improvement of pulse wave velocity 24 weeks (~6 month) after intervention.

### Sample size calculation

Sample size was calculated with G^*^ (http://www.gpower.hhu.de) according to the primary endpoint criterion. According to our preceding cross-sectional study (12, 13, 40) a HRPF z-score of about −0.64 ± 0.9 (27th percentile) was averaged in the CHD children in comparison to the healthy reference. The study aims to improve HRPF to a value of 0.0 ± 1.0 (50th percentile) at the end of the intervention. With a power of 85% on a one-sided level of significance of 0.05 a sample size of 31 per group is necessary. Assuming a slight drop out of about 10% in total 70 children will be recruited and allocated randomly 1:1 in the two study arms.

### Statistical methods

Difference of the HRPF z-score for each participant will be compared between both study groups using an independent two-sample *t*-test (if normal distributed) or Mann–Whitney U-Test (if skewed). To calculate gender specific and group specific differences over time, repeated ANOVA measures (if normal distributed) or Friedman-test (if skewed) will be performed. In terms of drop-out an intention to threat analysis will be performed. Two-sided level of significance of 5% will be considered for this primary endpoint.

## Discussion

The objective of this RCT is to determine the effect on HRPF and other functional outcome measures, as well as the compliance of a web-based exercise intervention in children with CHD.

Several studies in CHD have shown that participation in a physical exercise-training program is safe and improves fitness (18, 41). Unfortunately, those exercise interventions are underutilized in children with CHD and fitness, as the primary endpoint, is mostly just determined as an improvement in peak oxygen uptake while interest in other functional measures is rare. Indeed, peak oxygen uptake may be the most important prognostic parameter for survival in patients with CHD (19–21) but it is controversial whether that importance already exists in children with CHD. The idea behind is the changing landscape of older patients with CHD that will develop acquired cardiovascular disease, such as hypertension and hyperlipidemia (42, 43). From the perspective of primary prevention, it is therefore more important to shape PA behavior early in life that yield health benefits later in life. Motor skill development across the childhood has proven to influence children's PA behavior beneficially later in life in many ways (44, 45). Therefore, the objective of this study refers to HRPF and other functional outcomes instead of solely peak oxygen uptake.

Several studies already have investigated the feasibility and effectiveness of a home-based exercise intervention for young and adult people with CHD (14, 18). The utility of exercise interventions for this target group is unchallenged, but in 21 studies only 621 subjects, 30 per study, were included (18). Those small sample sizes very often lead to missing results and weak generalizability. Moreover, supervised training interventions are difficult to schedule and to conduct because of the low prevalence of patients with CHD.

To overcome those problems this study uses a web-based solution for two reasons. First, to include more patients in particular from remote areas and second, to deliver and monitor the intervention more effectively. The proposed training stimulus of 3 session of 20 min per week result in a relatively low real training volume of 30 min per week because the other 30 min are recovery time. Most of the studies mentioned in the comprehensive review (18) had longer durations for one training session and an overall higher workload per week but were conducted for only 12 weeks. Since this study takes twice as long we decided not to occupy the patient with intensive training. Instead it is assumed that less workload throughout the week leads to a better long-term compliance. That is also the reason why the patient is free in performing its 3 sessions per week instead of sticking to a fixed schedule.

The biggest challenge is the monitoring of the home-base intervention. Indeed 10 of the 21 studies did not report participation rate. Commonly in home-based intervention that is done by training logs and/or regular phone calls—a method that is inappropriate and especially uneconomical these days because they are not manageable outside a clinical study. With the electronic reporting and the possibility of tracking when training session were streamed it is much easier to follow compliance. In addition, automated messages can be sent to the patients to remind him/her of the execution and vice versa the supervisor when a training progress is missing. Nevertheless, it cannot entirely ruled out that the patients cheat in reporting. Consumer friendly wearable technology is a promising prospect in dealing with that issue but currently applications that are easy to use and connected to a central server are missing (46).

Web-based or eHealth PA interventions in patients with CHD are virtually not existent. Only the PReVaiL (23, 24) trial tried to assess benefits and harms of a tailored eHealth intervention with education and individual counseling in adolescents with CHD. Unfortunately, no change in oxygen uptake, PA and health-related quality of life occurred 52 weeks after the educational and motivational intervention. However, the most frustrating point was the low compliance to the intervention, which clearly outlined the vulnerability of those web-based approaches. Therefor our web-based intervention will contain exercise videos for a maximum of 24 weeks to minimize the number of drop-out. Further each exercise session consists of 20 different exercises that won't take more than 20 min. This means the amount of exercise is right at a level where changes in HRPF still can be expected but former inactive people aren't overstrained with an excessive exercise amount. Nevertheless, eHealth provides easy and wide reachability by low cost. Strategies have to be developed and evaluated to use this technique to maintain and improve the clinical care in this growing cohort of patients with CHD. This study aims to understand feasibility and compliance of those web-based studies.

## Author contributions

MM, A-LH, LB, RO, PE, and JM were all involved in development of the study protocol. JM prepared the initial draft of the manuscript. MM and AH set up the database infrastructure for the intervention. All authors read, contributed to editing, and approved the final manuscript.

### Conflict of interest statement

The authors declare that the research was conducted in the absence of any commercial or financial relationships that could be construed as a potential conflict of interest.

## Abbreviations

CHD: congenital heart disease
cIMT: Carotid Intima-media Thickness
HRPF: heat-related physical fitness
HRQoL: Health related Quality of Life
PWV: pulse wave velocity
RCT: randomized controlled trial.
